# Application of a 3D Bioprinted Hepatocellular Carcinoma Cell Model in Antitumor Drug Research

**DOI:** 10.3389/fonc.2020.00878

**Published:** 2020-06-03

**Authors:** Lejia Sun, Huayu Yang, Yanan Wang, Xinyu Zhang, Bao Jin, Feihu Xie, Yukai Jin, Yuan Pang, Haitao Zhao, Xin Lu, Xinting Sang, Hongbing Zhang, Feng Lin, Wei Sun, Pengyu Huang, Yilei Mao

**Affiliations:** ^1^Department of Liver Surgery, Peking Union Medical College (PUMC) Hospital, PUMC & Chinese Academy of Medical Sciences (CAMS), Beijing, China; ^2^State Key Laboratory of Medical Molecular Biology, Department of Physiology, Institute of Basic Medical Sciences, CAMS and School of Basic Medicine, PUMC, Beijing, China; ^3^Biomanufacturing Center, Department of Mechanical Engineering, Tsinghua University, Beijing, China; ^4^Biomanufacturing and Rapid Forming Technology Key Laboratory of Beijing, Beijing, China; ^5^Overseas Expertise Introduction Center for Discipline Innovation, Tsinghua University, Beijing, China; ^6^Department of Mechanical Engineering, Drexel University, Philadelphia, PA, United States; ^7^School of Life Science and Technology, ShanghaiTech University, Shanghai, China; ^8^CAS Center for Excellence in Molecular Cell Science, Chinese Academy of Sciences, Shanghai, China

**Keywords:** 3D bio-printing, liver, HCC model, drug screening, anti-tumor drug development

## Abstract

The existing *in vitro* models for antitumor drug screening have great limitations. Many compounds that inhibit 2D cultured cells do not exhibit the same pharmacological effects *in vivo*, thereby wasting human and material resources as well as time during drug development. Therefore, developing new models is critical. The 3D bioprinting technology has greater advantages in constructing human tissue compared with sandwich culture and organoid construction. Here, we used 3D bioprinting technology to construct a 3D model with HepG2 cells (3DP-HepG2). The biological activities of the model were evaluated by immunofluorescence, real-time quantitative PCR, and transcriptome sequencing. Compared with the traditional 2D cultured tumor cells (2D-HepG2), 3DP-HepG2 showed significantly improved expression of tumor-related genes, including ALB, AFP, CD133, IL-8, EpCAM, CD24, and β-TGF genes. Transcriptome sequencing analysis revealed large differences in gene expression between 3DP-HepG2 and 2D-HepG2, especially genes related to hepatocyte function and tumor. We also compared the effects of antitumor drugs in 3DP-HepG2 and 2D-HepG2, and found that the large differences in drug resistance genes between the models may cause differences in the drugs' pharmacodynamics.

## Introduction

An important tool in the screening of antitumor drugs is cytological studies, which usually include 2D planar cultures. However, the differences in conditions between planar cultures and *in vivo* environments are significant, with the expression of many key genes lost during the culture process (1). Moreover, many compounds that inhibit 2D cultured cells do not exhibit the same pharmacological effects *in vivo*, resulting in waste of large amounts of human and material resources as well as time during drug development. An increasing number of researchers are recognizing that 3D culture can, to some extent, bridge the gap between planar cultured cells and *in vivo* experiments, thus improving the success rate of drug development and reducing research costs before clinical trials (2).

Sandwich culture and organoid construction are widely used 3D culture methods. Sandwich culture and organoids overcome many limitations of 2D planar cultures, but they still have important limitations. Sandwich culture cells still grow in a plane and do not establish a spatial structure with each other, lacking interaction between cells. Owing to the physical properties of Matrigel, structural collapse occurs after a short period of *in vitro* culture. Long-term pharmacodynamic studies cannot be performed using this method (3). Moreover, the organoids must be cultured by stem cells through a complex induction process, and research using this system is complicated. In addition, the culture system requires various expensive growth factors and small-molecule compounds, resulting in high cost of the culture process. More importantly, owing to the manner of suspension culture *in vitro*, the diameters of the induced organoids vary, and the pharmacodynamic results obtained in drug screening could thus be undependable.

Hence, there remains an unmet need for a more appropriate *in vitro* tumor model for drug screening. 3D bioprinting has been reported to be a promising method for developing complex cancer cell models that can recapitulate the tumor microenvironment and drug response (4). Our research team previously constructed the first *in vitro* model of cervical cancer using 3D printing technology (5) and conducted preliminary biological function measurements and pharmacodynamic research. We also previously used a 3D bioprinting method to construct a human liver model that shows long-term maintenance of good liver function *in vitro* and can significantly prolong the lifespan of mice with liver failure after transplantation. This study indicates important potential applications of 3D bioprinting technology in liver-related biomedical fields (this manuscript is being reviewed). Studies have established 3D bioprinting as a convenient, efficient, economical, and easy-to-standardize operation of cutting-edge technology (5–8). Although current research on 3D printing focuses on the optimization of printing processes, selection of bio-inks, and evaluation of cell survival status, comprehensive and in-depth biological function evaluation and drug testing of 3D bioprinted tumor models are lacking.

To address the potential value of 3D printed tumor models for drug research, we established a 3D model of liver cancer composed of 3D bioprinted HepG2 cells and gelatin/alginate, and conducted a comprehensive comparison of these 3D bioprinted cells with 2D cultured cells. We evaluated differences in the two culture models and the effects of antitumor drugs in both models. Our findings may provide a basis for the application of 3D bioprinted tumor models in drug development research.

## Materials and Methods

### Cell Culture

HepG2 cells were purchased from the Cell Center of the Chinese Academy of Medical Sciences (Beijing, China). The cells were cultured in high-glucose Dulbecco's modified minimum essential medium (H-DMEM; Gibco, Logan, USA), supplemented with 10% fetal bovine serum (Gibco), 1% non-essential amino acid solution (Gibco), 1% penicillin G and streptomycin (Gibco), 1% glutamax (Gibco), 5 μg/ml insulin (Sigma, Saint Louis, USA), and 5 × 10^−5^ mol/L hydrocortisone hemisuccinate (Sigma). Cells were cultured in a 5% CO_2_ incubator at 37°C and passaged using trypsin (0.25%; Invitrogen, Carlsbad, USA) after reaching ~80% confluence. The culture medium was replaced every other day.

### Construction of the 3D Bioprinted HepG2 Model

A 3D cell printer (SPP1603) made by SUNP Co. was used to fabricate the *in vitro* liver cell model following a previously established method (9). Briefly, HepG2 cells were harvested and prepared as a suspension in a culture medium. The cell suspension and 4% sodium alginate solution were mixed at a volume ratio of 2:1. The mixture was incubated at 37°C for 5 min and then mixed with 20% gelatin solution at the indicated volume ratios, resulting in a final cell density of 5 × 10^5^/ml. One milliliter of the cell/biomaterial mixture was drawn into a sterilized syringe with a 23 G needle and set in a 3D printer at a controlled temperature. The temperatures of the nozzle and forming chamber were 20°C and 4°C, respectively. Petri dishes (35 mm in diameter) were pre-coated with 0.0125% (w/v) poly-L-lysine (P8920; Sigma-Aldrich) to collect the printed structures. The 3DP-HepG2 model was then fabricated by forced extrusion at a 150 mm^3^/min extrusion speed in a layer-by-layer fashion. The 3DP-HepG2 model was later immersed in 100 mM calcium chloride solution for 3 min to crosslink the sodium alginate and then transferred into 3 mL of fully supplemented H-DMEM. The medium was changed every 2 days.

### Cell Survival

Cell survival in the 3DP-HepG2 model was evaluated immediately after printing to assess the effect of the manufacturing process, particularly the hydrogel composition and temperatures of the nozzle and forming space, on cell viability. A fluorescent live/dead assay was performed to determine cell survival. Briefly, a mixture of calcein-AM (1 μmol/L; Sigma) and PI (2 μmol/L; Sigma) was prepared and passed through a 0.22-μm filter prior to staining. The 3DP-HepG2 model was gently washed with phosphate buffered saline after crosslinking and immediately incubated in a calcein-AM/PI mixture for 15 min at 20–25°C in the dark. After incubation, the 3DP-HepG2 model was gently washed with phosphate buffered saline and observed under a laser scanning confocal microscope (C2/C2si; Nikon, Tokyo, Japan). Five random fields were captured for each sample, and cells in five samples were counted using ImageJ. Cell viability was calculated by counting the number of cells as follows: (live cells/total cells) × 100%.

### Cell Morphology Imaging and Analysis

The cell morphology of 3DP-HepG2 was examined using an inverted optical microscope at culture days 0, 1, 3, 5, 7, and 10. Cell diameters were measured using a microscopic image processing software.

### Cell Proliferation Assay

3DP-HepG2 cells at culture days 0, 1, 3, 5, 7, and 10 were incubated in a mixture of culture medium and CCK-8 (Dojindo, Shanghai, China) at a volume ratio of 10:1. After 2 h of incubation at 37°C, fluorescence of the culture medium at 630 nm with 450 nm excitation was detected (Model 680; Bio-Rad, Berkeley, USA). A standard curve of fluorescence to a certain number of cells was established by incubation of HepG2 cells with CCK-8-containing culture medium in a six-well plate. The detected fluorescence of the 3D sample was then normalized to the cell number according to the standard curve.

### Protein and mRNA Expression

The expression of liver-related proteins and tumor signature-associated proteins and genes was evaluated by several methods. AFP secretion was measured using an ELISA kit (Alpha Fetoprotein ELISA; Abcam, Cambridge, UK); the protein expression of ALB, AFP, Ki67, and CYP3A4 was detected by immunofluorescence, and the mRNA expression of ALB, ATT, TTR, TAT, AFP, CD133, EpCAM, IL-8, CD24, MRP-1, MDR-1, ACBC1, LRP, BCRP, MRP2, and EGFR was detected by reverse-transcription polymerase chain reaction and real-time quantitative polymerase chain reaction. Antibody details and primer sequences are shown in Supplementary Tables 1, 2.

### RNA Sequencing and Bioinformation Analysis

Total mRNA was isolated using a TRIzol or RNeasy Mini Kit (QIAGEN, Dusseldorf, Germany) and reverse transcribed using a kit from Ambion (Austin, USA). *In vitro* transcription was performed using 1–5 ng cDNA as template, and RNA was reverse-transcribed into a sequencing library. Sequencing libraries were generated using the NEBNext® UltraTM RNA Library Prep Kit for Illumina® (NEB, USA) following the manufacturer's recommendations. Sequencing libraries were then sequenced on an Illumina HiSeq platform, and 125/150 bp paired-end reads were generated. Analysis of differentially expressed genes (DEGs) was performed using the DeSeq2 package. Genes with an adjusted *P* < 0.05 according to DESeq2 were assigned as DEGs. Gene Ontology (GO) and Kyoto Encyclopedia of Genes and Genomes (KEGG) enrichment analyses of DGEs were implemented using the cluster Profiler R package. GO terms and KEGG pathways with corrected *P* < 0.05 were considered significantly enriched by DEGs. A PPI network of DEGs was obtained from the STRING database; a confidence of 0.400 was chosen (version 11.0, https://string-db.org/). The PPI networks for upregulated genes were constructed using the Cytoscape3.6.1 software. The top 10 genes that were shared more than twice among the five types of centralities were defined as hub genes.

### Pharmacodynamic Evaluation of Antitumor Drugs

After 7 days of culture, there were (3.75 ± 0.19) × 10^5^ cells in 3DP-HepG2, and the same number of 2D-HepG2 cells were seeded in a 12-well plate dish. Both cell models were treated with different concentrations of cisplatin (Sigma) (0.01, 0.1, 1, 2.5, 5, 10, 20, and 100 μM), sorafenib (Selleck, Houston, USA) (0, 1, 0.625, 1.25, 2.5, 5, 10, 20, and 40 μM), or regorafenib (Selleck) (0, 1, 0.625, 1.25, 2.5, 5, 10, 20, and 40 μM) for 72 h. Cell growth was measured by the CCK8 assay, and dose-response curves were drawn using GraphPad 7.0.

### Statistical Analysis

Data are expressed as mean ± standard deviation. Statistically significant differences between the groups were determined using Student' s *t*-test. For all tests, a significance level of 5% (*P* < 0.05) was used.

## Results

### Construction of the 3D Bioprinted Hepatocellular Carcinoma Cell Model

We assembled HepG2 cells into a grid-like stereo structure using 3D printing technology. Compared with the traditional 3D culture method, this method is economical and efficient, with a high cell survival rate and uniform cell diameter.

**Figure 1:**
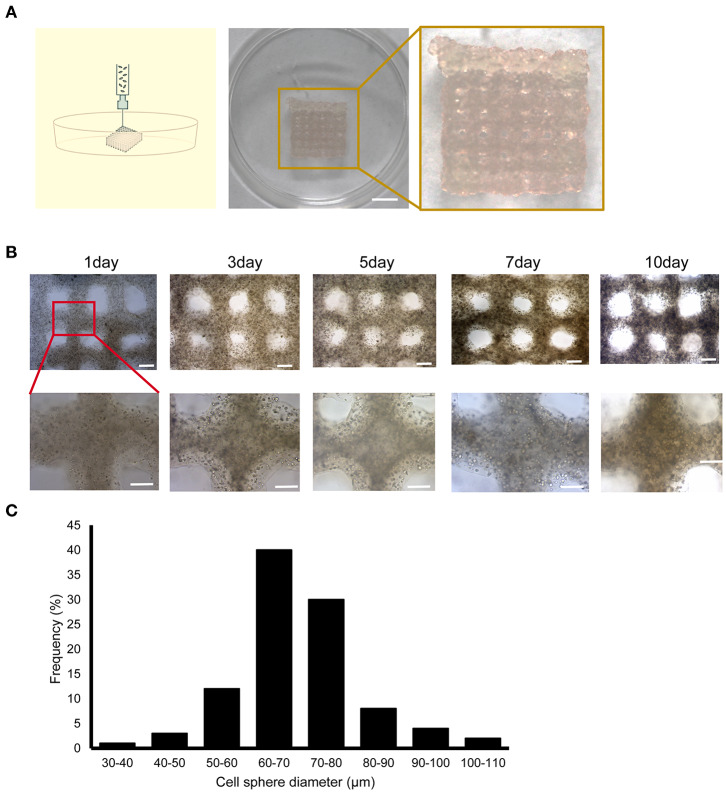
Construction of the 3D bioprinted liver cancer cell model. **(A)** Schematic illustration of the 3D cell-printing process (left) and the image of the 3DP-HepG2 model directly after printing (right). Scale bar: 50 mm. **(B)** Top view of the 3DP-HepG2 model on days 0, 3, 5, 7, and 10 after printing. Scale bar: 1 mm. Bottom row shows magnified view of insets (square). **(C)** Cell diameter distribution in the 3DP-HepG2 model at 10 days after printing.

In our previous work (7), we conducted investigations of bio-ink and printing processes. The results showed that gelatin and sodium alginate are excellent bio-inks with a stable structure, good biocompatibility, and low price. As shown in Figure 1A, the liver cancer cell model was printed according to a preset procedure through a 3D bioprinter provided by SUNP Company; this cell model was named 3DP-HepG2.

The printed 3DP-HepG2 model was cultured in H-DMEM with a medium change every 2 days. We observed the continuous growth of cells in the structure, and found that the cell sphere diameter gradually increased during culture. The 3DP-HepG2 structure remained clear and stable (Figure 1B). At 10 days after printing, the diameter of the cell spheres in the structure was examined. As shown in Figure 1C, ~70% of the cell spheres had diameters between 60 and 80 μm.

**Figure 2:**
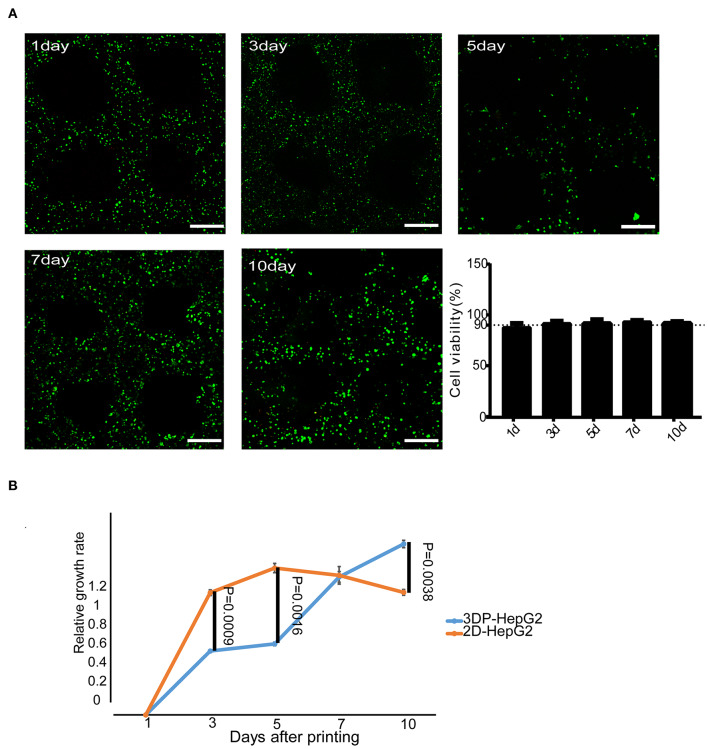
Cell survival and proliferation in the 3D bioprinted liver cancer cell model. **(A)** Cell viability at different times after printing. Representative live-dead staining images of 3DP-HepG2 structures at days 1, 3, 5, 7, and 10 after printing. Live and dead cells were labeled with calcein-AM (green) and PI (red), respectively. Scale bar: 300 μm. Histogram of cell viability at different times after printing **(B)** Proliferation rates of 3DP-HepG2 and 2D-HepG2 cells at different time points.

We used calcein-AM/PI staining to detect cell viability in the 3DP-HepG2 model. Cell viability was stable above 90% during the *in vitro* culture of 3DP-HepG2 (Figure 2A). We next compared the cell proliferation of 3DP-HepG2 and 2D cultured HepG2 (2D-HepG2) cells by CCK-8 assays. The number of cells in3DP-HepG2 at 5, 7, 10, and 15 days after printing was (2.15 ± 0.24) × 10^5^, (3.75 ± 0.19) × 10^5^, (4.3 ± 0.16) × 10^5^, and (3.88 ± 0.28) × 10^5^, respectively.

Compared with that of 2D-HepG2 cells, the growth rate of 3DP-HepG2 cells was slower for 1–6 days; however, at 7 days, the proliferation rates were similar in both cultures, whereas at 10 days, the proliferation rate of 3DP-HepG2 cells was much higher than that of 2D-HepG2 cells (*P* = 0.0038) (Figure 2B). Therefore, we selected 7 days after printing as the time point for most functional examination, including protein expression, transcriptome analysis, and drug studies, as this time point was judged to be suitable for comparison of 3D printed cells with planar culture cells. Only when assessing the effect of culture time on the functional gene expression of cells were 5, 10, and 15 days after printing used as key time points.

### Liver-Related Protein and mRNA Expression

**Figure 3:**
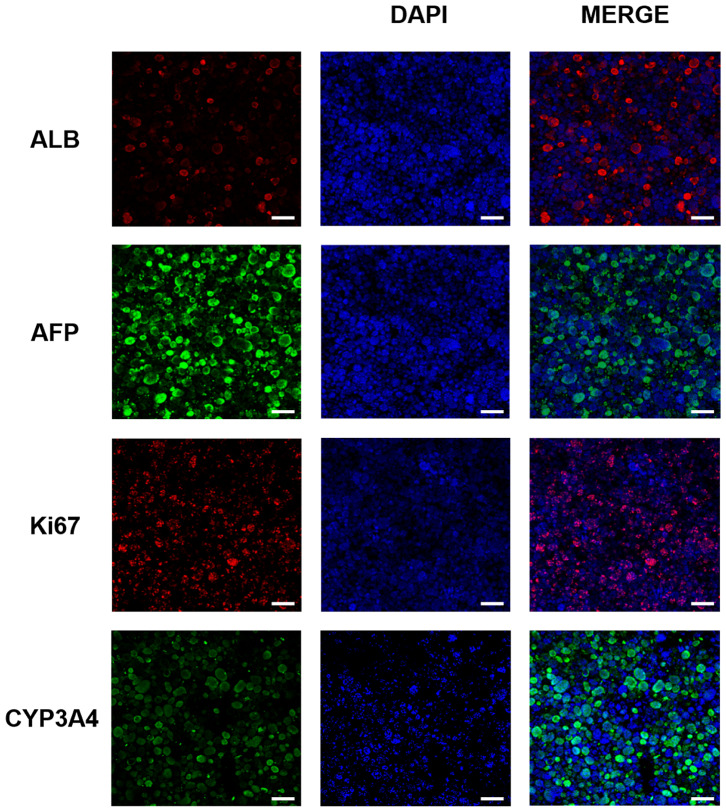
Liver-related protein expression in the 3D bioprinted liver cancer cell model. ALB, AFP, Ki67, and CYP3A4 protein expression in the 3DP-HepG2 model at 7 days after printing. Scale bar: 200 μm.

We examined the expression of various liver-associated proteins in the 3DP-HepG2 model by immunofluorescence. The results showed that ALB, AFP, Ki67, and CYP3A4 were expressed (Figure 3).

To investigate the mRNA expression of liver-related genes, we collected 2D-HepG2 cells in the logarithmic growth phase and 3DP-HepG2 model cells at 5, 10, and 15 days after 3D printing. We then extracted RNA for qRT-PCR. The mRNA expression levels of liver function-related genes, such as ALB, AAT, TTR, TAT, CYP2D6, and CYP3A4, were higher in the 3DP-HepG2 model than in 2D-HepG2 cells. ALB and TTR mRNAs decreased over culture time, whereas AAT mRNA maintained a high expression level (Supplementary Figures 1A–F). The expression level of cytochrome is an important indicator of liver function (10). Both CYP2D6 and CYP3A4 mRNA levels in the 3DP-HepG2 model were higher than those in 2D-HepG2 cells, and the expression levels were the highest at 10 days of culture, reaching 15- and 9-fold higher than those in 2D-HepG2 cells, respectively, (Supplementary Figures 1E,F).

### Tumor-Related Protein and mRNA Expression

**Figure 4:**
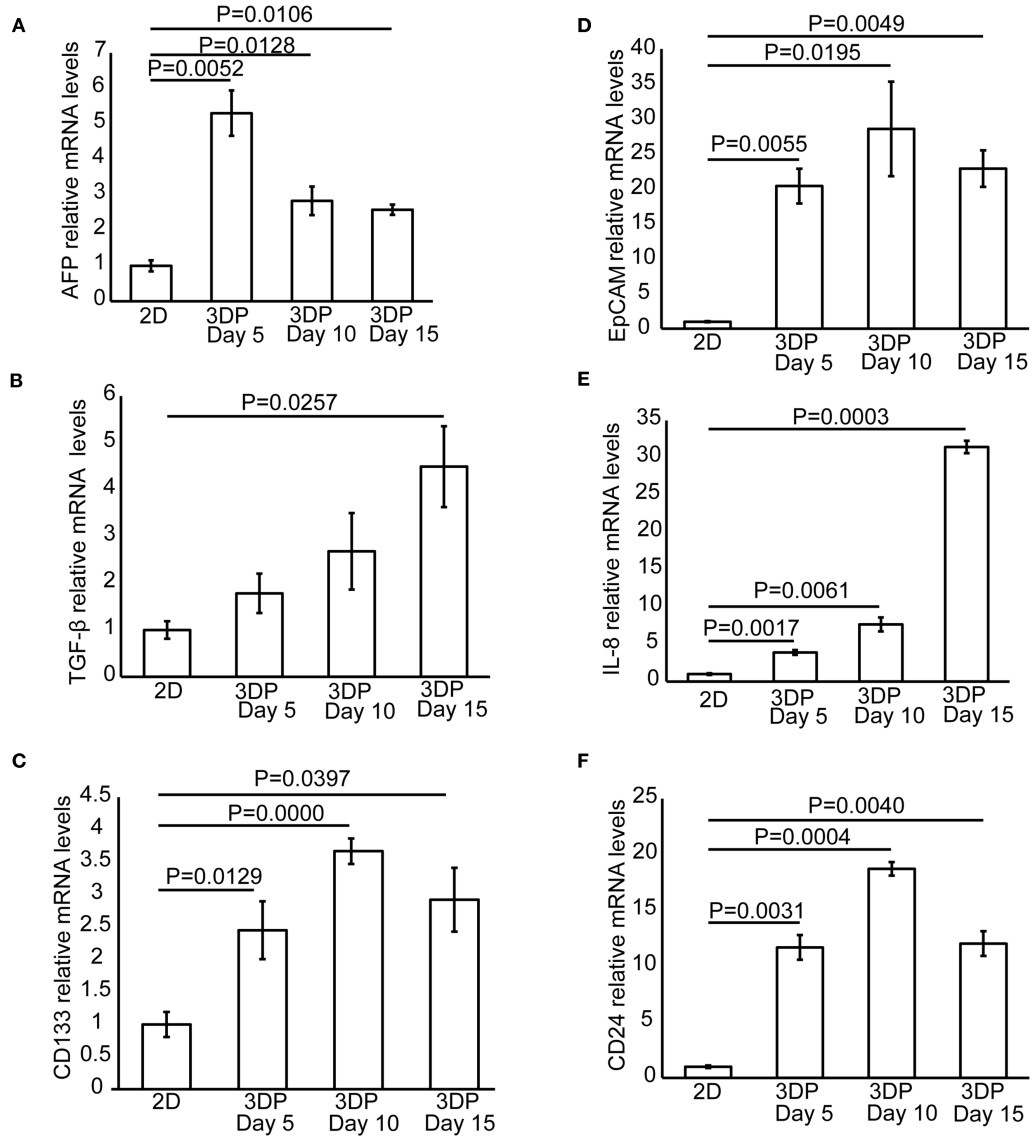
Tumor-related protein and mRNA expression in the 3D bioprinted liver cancer cell model. The mRNA expression of tumor-related genes, including **(A)** AFP, **(B)** TGF-β, **(C)** CD133, **(D)** EpCAM, **(E)** IL-8, and **(F)** CD24 in the 2D-HepG2 and 3DP-HepG2 models at 5, 10, and 15 days after 3D printing.

To detect the level of AFP protein secreted by HepG2 cells, we used ELISA to measure AFP levels in the supernatant of 3DP-HepG2 and 2D-HepG2 cells after 7 days of culture. The results are shown in Figure 4A. The concentration of AFP in the supernatant of the 3DP-HepG2 model was 996.3 ± 166.6 ng/10^6^ cell/day, which was much higher than that in the supernatant of 2D-HepG2 cells, at 663.3 ± 81.6 ng/10^6^ cells/day (*P* = 0.0387) (Supplementary Figure 2).

We also collected mRNA from 2D-HepG2 cells in the logarithmic growth phase and 3DP-HepG2 cells at 5, 10, and 15 days after 3D printing and evaluated the expression of tumor-related genes. The expression patterns of AFP mRNA in this assay were similar to those in the ELISA assay. In the 3DP-HepG2 model cultured for 5 days, the level of AFP mRNA was ~2.5-fold higher than that of 2D-HepG2 cells and ~3-fold higher than that of cells cultured for 10 and 15 days (Figure 4A). The expression level of TGF-β mRNA in the 3DP-HepG2 model was much higher than that in the 2D model and gradually increased over culture time, reaching 1.8-, 2.7-, and 4.5-fold higher than that in 2D-HepG2 cells at 5, 10, and 15 days, respectively, (Figure 4B). CD133 mRNA expression level was significantly higher in the 3DP-HepG2 model than in 2D-HepG2 cells by ~2.5-fold at 5 days, 3.7-fold at 10 days, and 3-fold at 15 days (Figure 4C). The expression level of EpCAM mRNA in the 3DP-HepG2 model was much higher than that in 2D-HepG2 cells by over 20-fold (Figure 4D). The expression level of IL-8 mRNA in the 3DP-HepG2 model was significantly higher than that in 2D-HepG2 cells, and increased with time. At 15 days after printing, IL-8 mRNA expression level was 31-fold higher than that in 2D-HepG2 cells (Figure 4E). The expression level of CD24 mRNA showed similar results; the expression level in the 3DP-HepG2 model was more than 10-fold higher than that in 2D-HepG2 cells (Figure 4F).

### Transcriptional Profiling of 3D-Printed Liver Cancer Model

**Figure 5:**
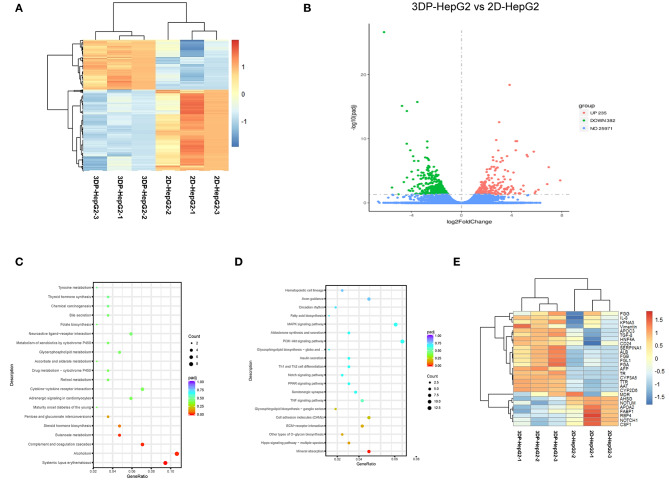
Transcriptional profiling characterization of 3D printed liver cancer cell model. **(A)** Heatmap of DEGs between the 3DP-HepG2 and 2D-HepG2 models. Rows represent genes, and columns represent samples. **(B)** Volcano plot showing 617 DEGs, including 235 significantly upregulated DEGs (red spots) and 382 significantly downregulated DEGs (green spots). KEGG pathway enrichment bubble chart of **(C)** significantly upregulated genes and **(D)** downregulated genes. The x–axis represents fold of enrichment and the y–axis represents KEGG–enriched terms. The size of the dot represents the number of genes under a specific term. The color of the dots represents adjustment. **(E)** The expression of liver cancer-specific genes in the 3DP-HepG2 and 2D-HepG2 models. The heatmap shows the expression of hepatocyte-related genes and tumor-related genes in the models. DEGs, differentially expressed genes; GO, Gene Ontology; KEGG, Kyoto Encyclopedia of Genes and Genomes.

We next performed mRNA sequencing to compare the transcriptional characterization of the 3DP-HepG2 and 2D-HepG2 models. Cluster analysis showed that the 3DP-HepG2 model had a unique gene expression profile (Figure 5A), which suggested that cells in the 3D and 2D culture models had different microenvironments. A total of 617 DEGs were identified, including 235 significantly upregulated DEGs and 382 significantly downregulated DEGs (Figure 5B). We conducted GO and KEGG pathway enrichment analyses to explore the functional characteristics of the DEGs. GO analysis results showed that the upregulated DEGs were significantly enriched in “extracellular matrix disassembly,” “blood microparticle,” “nucleosome, DNA packaging complex,” “CoA-ligase activity,” “sodium ion transmembrane transporter activity,” “C-acyltransferase activity,” and “fatty acid ligase activity” (Supplementary Figure 3A). For the downregulated DEGs, significant enrichment was observed in “the cellular response to zinc ion,” “viral entry into host cell,” “extracellular matrix organization,” “extracellular structure organization,” “proteinaceous extracellular matrix,” “extracellular matrix,” “endoplasmic reticulum lumen,” and “integrin binding” (Supplementary Figure 3B). KEGG analysis results showed that the upregulated DEGs were significantly enriched in “Systemic lupus erythematosus,” “Alcoholism,” “Complement and coagulation cascades,” and “Butanoate metabolism” (Figure 5C). Downregulated DEGs were significantly enriched in “mineral absorption,” “cell adhesion molecules,” and “TNF signaling pathway” (Figure 5D).

Hepatocyte-related genes and tumor-related genes are shown separately in Figure 5E. The expression levels of functional hepatocyte genes (ALB, AAT, TTR, HNF4A, CYP P450, and glycogen metabolism genes) were much higher in the 3DP-HepG2 model than in the 2D model cells, which indicated that HepG2 cells may reach further maturation in the 3D-printed model with better hepatocyte function. The differences in cell differentiation and cancer-related genes (AFP, NOTCH1, CSF1, NOTUM, TGFβ, and vimentin genes) implied different biological characteristics between cells cultured in the 3D and 2D models.

DEGs usually perform biological functions synergistically, and strong relationships were shown in PPI network analysis (Supplementary Figures 3C,D). The hub genes of the upregulated DEGs were mainly associated with hepatocyte function, which was consistent with the results of heatmap analysis. The hub genes of downregulated DEGs were involved in regulating cell apoptosis, proliferation, and differentiation. These hub genes led to differences between the 3DP-HepG2 and 2D-HepG2 models (Table 1).

**Table 1:** Hub genes of differentially expressed genes in the 3D-printed model compared with the 2D model.

**Upregulated DEGs**		**Downregulated DEGs**	
Hub gene	Function	Hub gene	Function
ALB	Liver function	NOTUM	Wnt inhibitor
APOA4	Lipid metabolism	NOTCH1	Cell apoptosis, proliferation, and differentiation
SERPINC1	Coagulation system	MATN3	Extracellular matrix
PLG	Coagulation system	MFGE8	Cell apoptosis, cell proliferation
GC	Vitamin D metabolism	CSF1	Cell apoptosis, cell proliferation, and cell differentiation
APOC3	Lipid metabolism	LAMB1	Extracellular matrix
VTN	Cell migration, cell proliferation	LGALS1	Cell apoptosis, cell proliferation, and cell differentiation

### Effects of Antitumor Drugs on the 3DP-HepG2 Model

**Figure 6:**
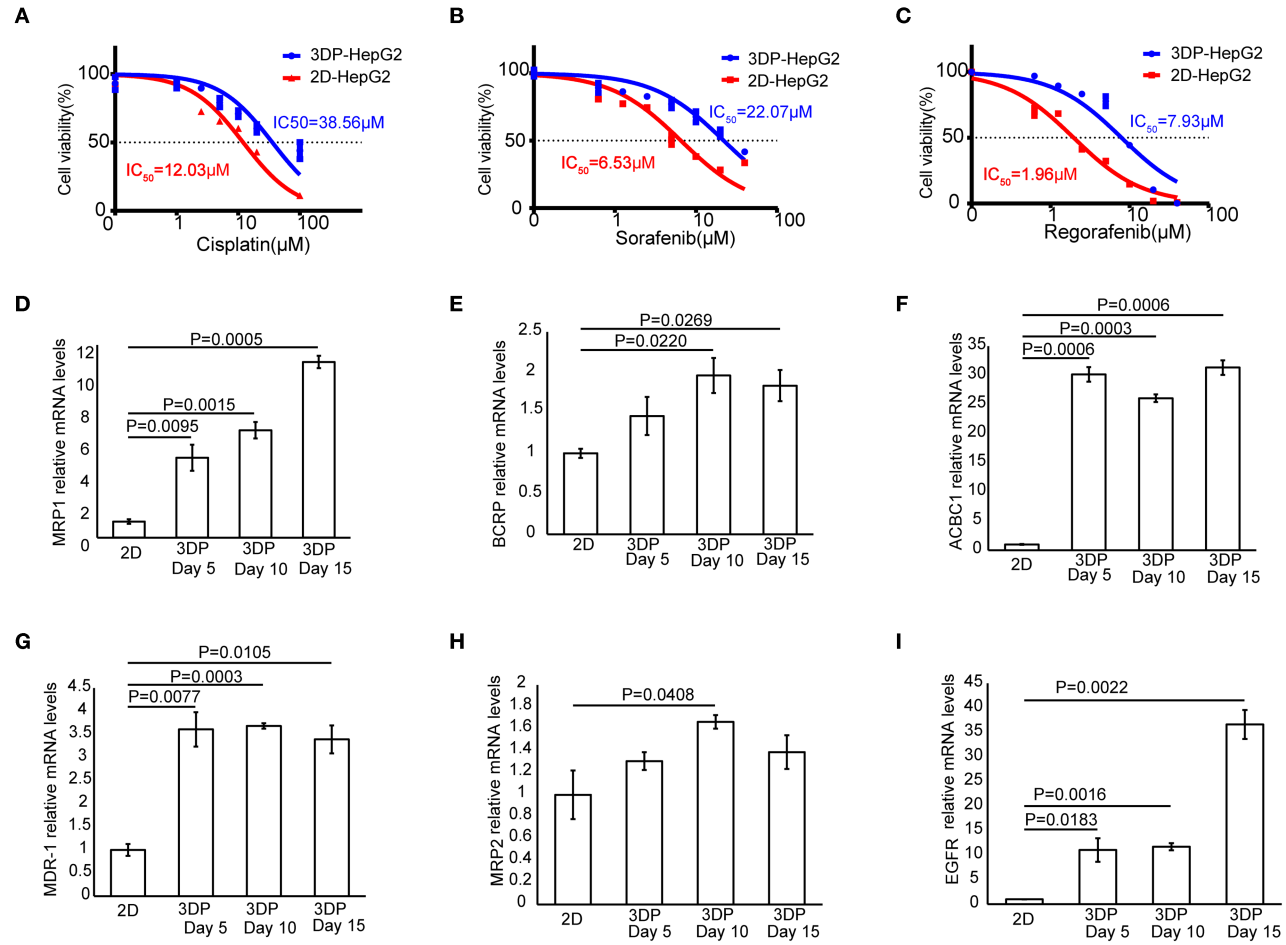
Characteristics of drug metabolism in the 3D bioprinted liver cancer cell model. Dose-effect curves of cisplatin **(A)**, sorafenib **(B)**, and regorafenib **(C)** in the 3DP-HepG2 and 2D-HepG2 models after 72 h of treatment. The mRNA expression of drug resistance genes in the 2D-HepG2 and 3DP-HepG2 models at 5, 10, and 15 days after 3D printing. **(D)** MRP1, **(E)** BCRP, **(F)** ACBC1, **(G)** MDR-1, **(H)** MRP2, and **(I)** EGFR mRNAs.

To test the response of the 3DP-HepG2 model to antitumor drugs, we treated the 3DP-HepG2 and 2D-HepG2 models with cisplatin, sorafenib, and regorafenib for 72 h. The IC_50_ values of cisplatin, sorafenib, and regorafenib in the 3DP-HepG2 model were significantly higher than those in the 2D-HepG2 model (38.56 vs. 12.03 μM, 22.07 vs. 6.53 μM, and 7.93 vs. 1.96 μM, respectively) (Figures 6A–C).

To explain this finding, we examined the expression of multiple drug resistance genes and autophagy-related genes (11, 12), including MRP1, MDR-1, ACBC1, BCRP, MRP2, EGFR, Beclin-1, LC3A, LC3B, and Atg5, among others. qRT-PCR revealed large differences in drug resistance gene levels in the two models. In the 3DP-HepG2 model, MRP1, ACBC1, MDR-1, and EGFR mRNA levels increased significantly at every time point compared with those in the 2D model (Figures 6D,F,G,I), whereas BCRP mRNA level significantly increased at 10 and 15 days after printing (Figure 6E) and MRP2 mRNA level significantly increased only at 10 days after printing (Figure 6H).

The expression of autophagy-related genes also significantly increased in the 3DP-HepG2 model compared with that in the 2D-HepG2 model (Supplementary Figure 4). Beclin-1 mRNA expression in the 3DP-HepG2 model was significantly higher than that in 2D-HepG2 cells by 8.0-, 5.2-, and 4.9-fold at 5, 10, and 15 days, respectively. The expression level of LC3A mRNA in the 3DP-HepG2 model increased over time, and the expression level was 10.7-fold higher than that in 2D cultured cells at 15 days after printing. The expression level of LC3B mRNA showed similar results, with 35-fold higher expression in the 3DP-HepG2 model at 10 days than that in 2D-HepG2 cells. The expression of Atg5 mRNA in the 3DP-HepG2 model was maintained at a high level, ~9-fold higher than that in 2D-HepG2 cells.

## Discussion

Cell-based drug efficacy and toxicity assays of lead compounds are essential before application of these compounds in clinical trials. Current drug screening in *in vitro* models mainly relies on 2D cultured cells. However, 2D cultured cells lead to a high rate of failure of drugs entering clinical trials because they fail to mimic the *in vivo* microenvironment (13–15). Therefore, a more representative human tumor model in the preclinical phase of drug development is required (16, 17). In this study, we developed a novel 3D tumor model using 3D bioprinting. Compared with the standard 2D models, 3D bioprinted tumor models offer a tumor microenvironment that is more similar to *in vivo*, including the extracellular matrix, specific size, spatial distribution, and geometry, which affect the functional state and interaction of tumor cells (15). Indeed, we found that 3D bioprinted tumor models showed higher levels of tumor-related genes and liver-related genes, which was similar to the expression patterns observed in human tumors (18, 19). Moreover, 3D bioprinted tumor models showed a better correlation to human clinical trials in terms of response to chemotherapeutic agents, which will be helpful in accelerating drug development processs and reducing the failure risks and costs of drug screening.

The tumor model in our method was generated by extrusion-based bioprinting, which is simple, easy to operate, convenient, fast, and economical. The 3DP-HepG2 model had a stable structure and good material exchange environment, and did not require specific factors. Furthermore, this model showed high cell survival rate. The cell cluster in the 3D model had a relatively large diameter and sufficient growth space and can be cultured for a long time, which is advantageous for long-term pharmacodynamic studies. In addition to extrusion-based bioprinting, there are other methods of 3D bioprinting, mainly droplet-based bioprinting, laser-based bioprinting, and photocuring-based bioprinting (20). Droplet-based bioprinting can be used to construct models with high resolution, but it usually causes damage to cells. Moreover, using this method, it is difficult to print biomaterials with high viscosity, which affects their structural stability. Laser-based bioprinting has been reported to generate tumor cell models. Laser-based bioprinting is a nozzle-free technology that avoids cell mechanical injury and can even manipulate a single cell. Kingsley et al. (21) reported that they achieved spatial and size control of tumor spheroids using laser-based 3D bioprinting, which indicates its potential to construct delicate and homogeneous tumor models. However, compared with the extrusion-based bioprinting used in our study, laser-based bioprinting causes more damage to cells. More importantly, it is expensive and difficult to operate. Photocuring-based bioprinting has the same problems and needs further development.

In our examination of the biological behavior of tumor cells, we found that compared with 2D-HepG2 cells, the 3DP-HepG2 model showed significantly increased levels of various liver function-related proteins and genes, as well as proteins and genes involved in the proliferation, metastasis, drug resistance, antitumor immunosuppression, and epithelial to mesenchymal transition of tumor cells. The higher levels of ALB and AAT mRNAs in 3DP-HepG2 model than those in planar cultured cells indicated that 3D printed tumor models had stronger liver function. The stem cell markers CD133 and EpCAM were significantly increased in 3D printed tumor cells, which implied that the tumor cells in the 3D printed model were superior to planar cultured cells in terms of invasion, metastasis, drug resistance, and recurrence (22–24). Furthermore, we found that immunosuppression was enhanced in the 3D printed tumor models because of the higher levels of IL-8 and CD24.

We also identified key features of the 3DP-HepG2 model from a transcriptome perspective. The 3DP-HepG2 model had a distinct transcriptional expression profile, which may be due to its unique microenvironment and functional differences compared with those of 2D cultured cells. The 3DP-HepG2 model showed increased expression of genes involved in liver functions, such as protein synthesis, lipid metabolism, and glycogen metabolism, suggesting that the 3D-printed microenvironment may support further differentiation of HepG2 cells. However, the specific genes involved in this phenomenon are currently unknown. Identifying the key genes causing the differences between the cell characteristics and environments of 3DP-HepG2 and 2D-HepG2 models will be beneficial to understanding how the microenvironment of the 3D printed model affects cell characteristics. We identified some hub genes involved in cell differentiation, especially NOTUM, which functions as a Wnt inhibitor (25). The Wnt pathway plays an important role in embryonic development, cell differentiation, and tumorigenesis (26). These results suggested that Wnt pathway activation may be involved in the differences between the environments of 3DP-HepG2 and 2D-HepG2.

The differences in the expression levels of various genes reflect the difference in the biological activities of tumor cells in the two models, and this was confirmed by the assay of the antitumor effects of several drug treatments. We found that the IC_50_ values of the three tested drugs were much higher in the 3DP-HepG2 model than in the 2D-HepG2 model. The IC_50_ values obtained in the 3DP-HepG2 model were closer to the effective blood concentration of the drugs in the human body (27–29). We also examined several common drug resistance genes and autophagy-related genes, and found that the expression levels of ACBC1, MDR-1, MRP1, and EGFR significantly increased in the 3DP-HepG2 model, which may explain the different effects of drugs in the two cell models. When suffering from drug stress, tumor cells evolve through certain mechanisms. For example, tumor cells can enhance drug efflux by upregulating MDR-1 genes (30). Moreover, they can gradually adapt to drugs by enhancing autophagy (31). Therefore, it is speculated that the expression of drug resistance genes and autophagy-related genes will increase after antitumor drug treatment.

However, there are some limitations to our study. Above all, tumors contain different types of cells, but only tumor cells were included in our 3D printed model. Our group is also conducting studies on complex tumor structures, including tumor cells, endothelial cells, immune cells, and other cellular components. Because of the lack of feasible methods for 3D cultured cells, we compared the biological characteristics of a 3D printed model and 2D culture by assessing functional gene expression, instead of biological phenotypes such invasion and metastasis.

## Conclusions

The tumor-associated biological activity of HepG2 cells in the liver cancer tumor model constructed using 3D bioprinting technology was higher than that of the traditional planar culture cells. Using a 3DP-HepG2 model for drug research may produce pharmacodynamic results that are closer to the actual conditions *in vivo*. This 3D bioprinted tumor model has broad application prospects in drug development.

## Data Availability Statement

This data can be found here: the NCBI Sequence Read Archive (https://www.ncbi.nlm.nih.gov/sra)(PRJNA626409).

## Author Contributions

LS, HY, YW, XZ, and BJ: methodology, investigations, formal analysis, data curation writing-original, and draft preparation. FX, YJ, YP, and HZhao: data analysis, interpretation, and formal analysis. XL, XS, and HZhan: writing-review and editing. FL: funding acquisition, supervision and writing-review, and editing. YM, PH, and WS: study conceptualization, project administration, writing-review and editing, and final approval of manuscript

## Conflict of Interest

The 3D bioprinter was provided by HEALTH Biomed Company. The authors declare that the research was conducted in the absence of any other commercial or financial relationships that could be construed as a potential conflict of interest. The HEALTH Biomed Company was not involved in the study design, collection, analysis, interpretation of data, the writing of this article or the decision to submit it for publication.

## Supplementary Material

Supplementary Figure 1 Liver-related mRNA expression in the 3D bioprinted liver cancer cell model. Expression of liver related-genes in the 2D-HepG2 model in the logarithmic growth phase and the 3DP-HepG2 model at 5, 10, and 15 days after 3D printing. **(A)** ALB, **(B)** AAT, **(C)** TTR, **(D)** TAT, **(E)** CYP2D6, and **(F)** CYP3A4 mRNAs.

Supplementary Figure 2 AFP expressionin 3D bioprinted liver cancer cell model. AFP levels in the supernatant of the 3DP-HepG2 and 2D-HepG2 models after 7 days of culture.

Supplementary Figure 3 Protein-protein interaction network of DEGs between the 3DP-HepG2 and 2D-HepG2 models. GO cluster plot showing a histogram of the clustering of the expression spectrum of significantly upregulated genes **(A)** and downregulated genes **(B)**. Protein-protein interaction network showed correlations between differentially expressed protein-encoding genes, including **(C)** upregulated DEGs and **(D)** downregulated DEGs. DEGs, differentially expressed genes.

Supplementary Figure 4 Expression of autophagy-related genes in 3D bioprinted liver cancer cells. The mRNA expression of autophagy-related genes in the 2D-HepG2 and 3DP-HepG2 models at 5, 10, and 15 days after 3D printing. **(A)** Beclin-1, **(B)** LC3A, **(C)** LC3B, and **(D)** Atg5 mRNAs.

Supplementary Table 1 The antibodies for immunofluorescent.

Supplementary Table 2 The primers for qPCR.
