# Supplementary figures and images for: Application of a 3D Bioprinted Hepatocellular Carcinoma Cell Model in Antitumor Drug Research
Source: Front Oncol. 2020 Jun 3;10:878. doi: 10.3389/fonc.2020.00878 (PMC7283506; doi:10.3389/fonc.2020.00878)

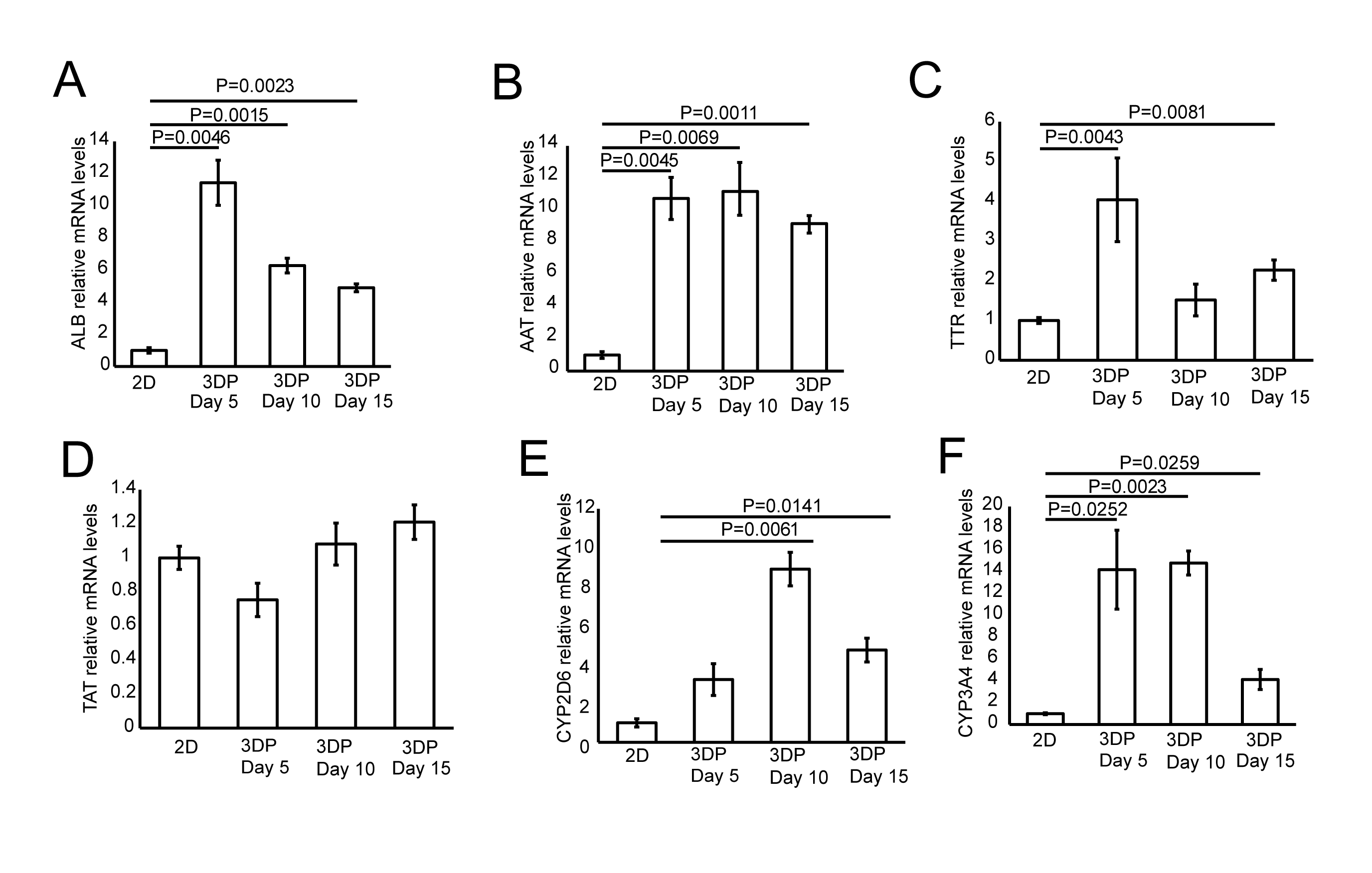

Supplement: Supplementary Figure 1 — Liver-related mRNA expression in the 3D bioprinted liver cancer cell model. Expression of liver related-genes in the 2D-HepG2 model in the logarithmic growth phase and the 3DP-HepG2 model at 5, 10, and 15 days after 3D printing. (A) ALB, (B) AAT, (C) TTR, (D) TAT, (E) CYP2D6, and (F) CYP3A4 mRNAs. [file Image_1.tif]

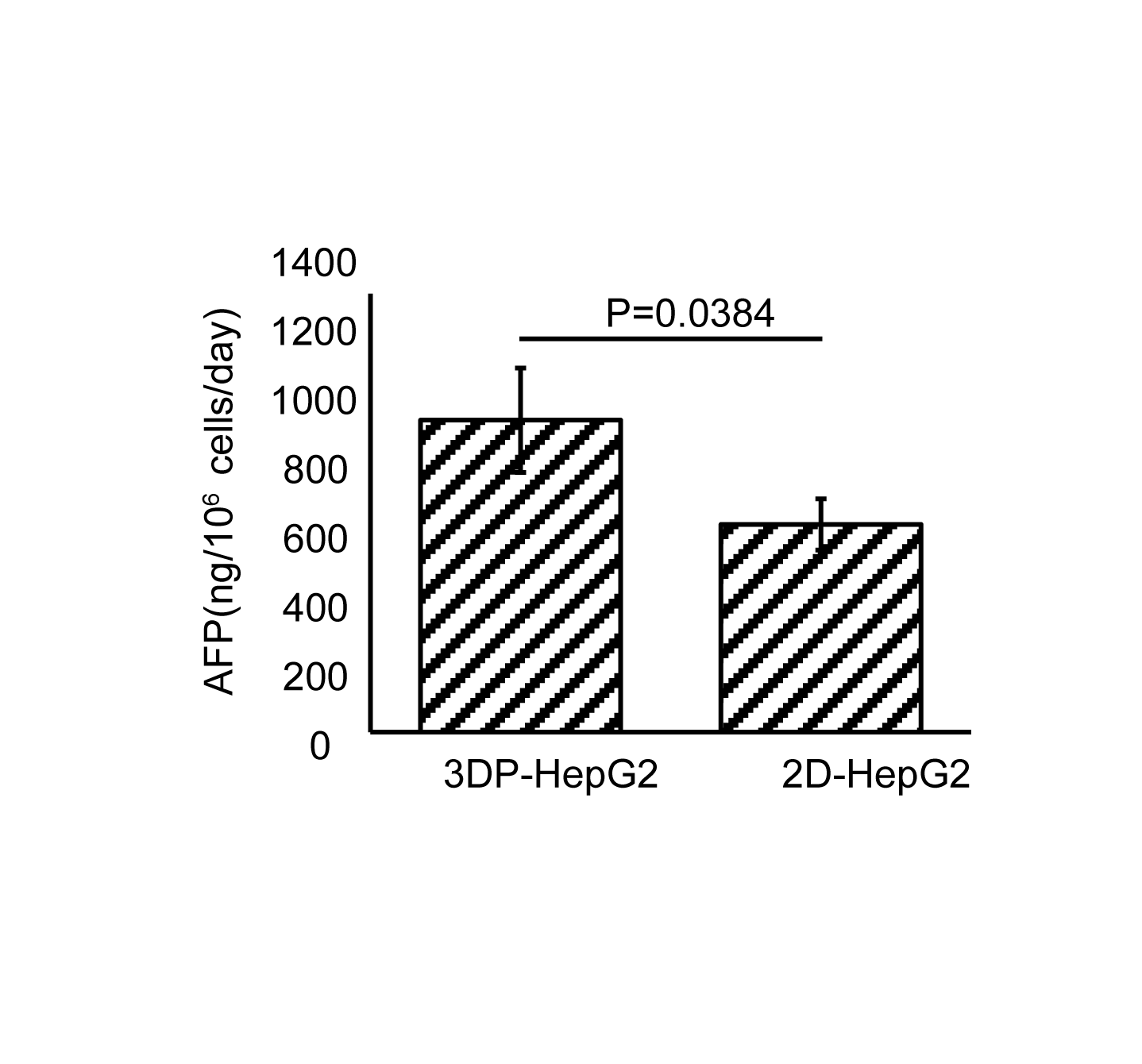

Supplement: Supplementary Figure 2 — AFP expressionin 3D bioprinted liver cancer cell model. AFP levels in the supernatant of the 3DP-HepG2 and 2D-HepG2 models after 7 days of culture. [file Image_2.TIF]

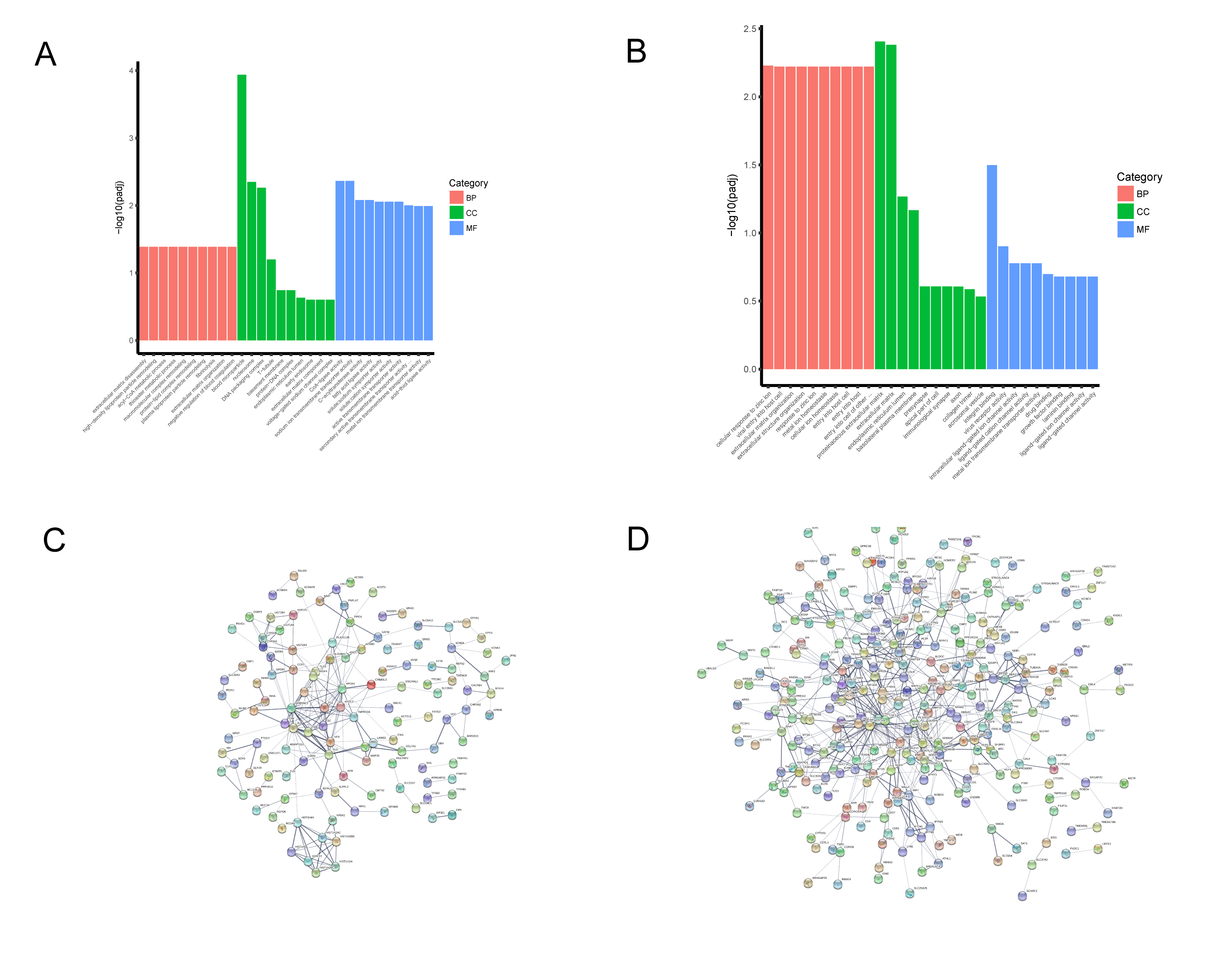

Supplement: Supplementary Figure 3 — Protein-protein interaction network of DEGs between the 3DP-HepG2 and 2D-HepG2 models. GO cluster plot showing a histogram of the clustering of the expression spectrum of significantly upregulated genes (A) and downregulated genes (B). Protein-protein interaction network showed correlations between differentially expressed protein-encoding genes, including (C) upregulated DEGs and (D) downregulated DEGs. DEGs, differentially expressed genes. [file Image_3.TIF]

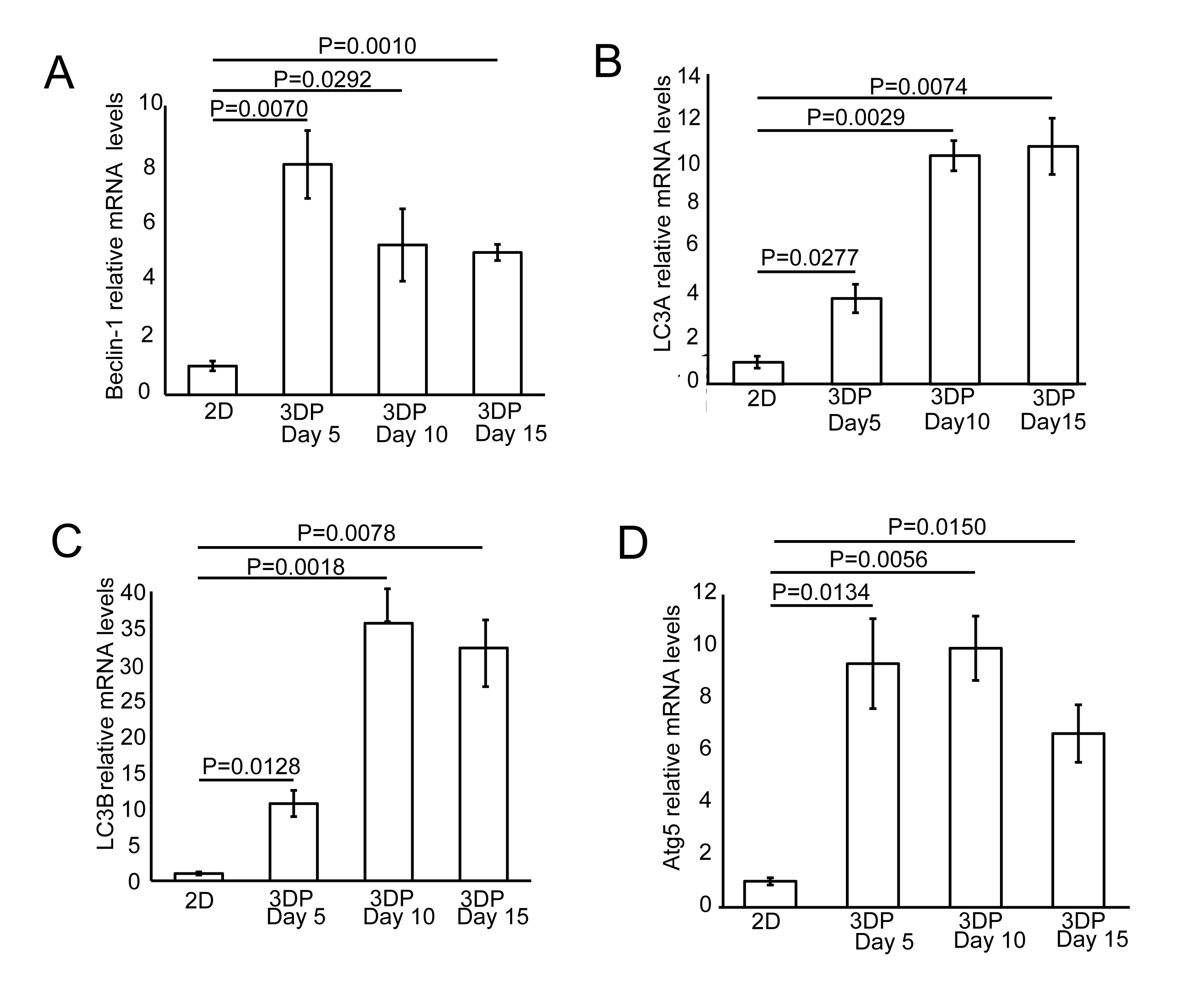

Supplement: Supplementary Figure 4 — Expression of autophagy-related genes in 3D bioprinted liver cancer cells. The mRNA expression of autophagy-related genes in the 2D-HepG2 and 3DP-HepG2 models at 5, 10, and 15 days after 3D printing. (A) Beclin-1, (B) LC3A, (C) LC3B, and (D) Atg5 mRNAs. [file Image_4.TIF]
