# Supplementary material for: Application of a 3D Bioprinted Hepatocellular Carcinoma Cell Model in Antitumor Drug Research
Source: Front Oncol. 2020 Jun 3;10:878. doi: 10.3389/fonc.2020.00878 (PMC7283506; doi:10.3389/fonc.2020.00878)
Supplement: Supplementary Table 1 — The antibodies for immunofluorescent. [file Data_Sheet_1.docx]

**Supplementary table 1. The antibodies for immunofluorescent**

| **Antibodies** | **Concentration** | **Source** |
| --- | --- | --- |
| Goat anti--human ALBUMIN | 1:200 | Bethyl |
| Rabbit anti-human alpha 1 Fetoprotein | 1:250 | Abcam |
| Mouse anti-human CYP3A4 | 1:50 | Santa cruz |
| Rabbit anti-human Ki 67 | 1:500 | Abcam |
| Goat anti-Rabbit (Alexa Fluor® 594) | 1:500 | Abcam |
| Goat anti-Mouse(Alexa Fluor® 488) | 1:500 | Abcam |
| Donkey anti-Goat (Alexa Fluor® 594) | 1:500 | Abcam |

**Supplementary table 2. The primers for qPCR**

| **Gene** | **Forward (5’-3’)** | **Reverse (5’-3’)** |
| --- | --- | --- |
| MRP1 | TCGCCATTGAAATAGCTGCGGC | CGCATAGTGGATGGCTTTCAGC |
| MRP2 | AGCGTCCTCTGACACTCG | GGCATCTTGGCTTTGACT |
| MDR-l | TTGCTGCTTACATTCAGGTTTCA | AGCCTATCTCCTGTCGCATTA |
| ABCBl | TGACATTTATTCAAAGTTAAAAGCA | TAGACACTTTATGCAAACATTTCAA |
| LRP | ACAACT ACT GCG TGA TTC TC | TCA GCA TGT AGG TGC TTC CA |
| BCRP | GTTTCAGCCGTGGAAC | CTGCCTTTGGCTTCAAT |
| Atg5 | TCTGGATGGGATTGCAAAATG | TTTCTTCTGCAGGATATTCCATGA |
| Beclin-1 | CTGGACACGAGTTTCAAGATCCT | TGTGGTAAGTAATGGAGCTGTGAGTT |
| LC3A | TCCCGGACCATGTCAACAT | ACCATGCTGTGCTGGTTCAC |
| LC3B | ACCATGCCGTCGGAGAAG | ATCGTTCTATTATCACCGGGATTTT |
| CD133 | TCTGGGTCTACAAGGACTTTCC | GCCCGCCTGAGTCACTAC |
| EPCAM | AAT CGT CAA TGC CAG TGT ACT T | TCT CAT CGC AGT CAG GAT CAT AA |
| EGFR | TAACAAGCTCACGCAGTTGG | GTTGAGGGCAATGAGGACAT |
| ALB | AGCATGGGCAGTAGCTCGCCT | AGGTCCGCCCTGTCATCAGCA |
| AAT | TATGATGAAGCGTTTAGGC | CAGTAATGGACAGTTTGGGT |
| TAT | GCATCCTATGTCGCACCC | TCAGCAACTAACCGCTCC |
| TTR | TGGGAGCCATTTGCCTCTG | AGCCGTGGTGGAATAGGAGTA |
| CYP2D6 | TGAAGGATGAGGCCGTCTGGGAGA | CAGTGGGCACCGAGAAGCTGAAGT |
| CYP3A4 | CAGGAGGAAATTGATGCAGTTTT | GTCAAGATACTCCATCTGTAGCACAGT |
| IL-8 | ACTGAGAGTGATTGAGAGTGGAC | AACCCTCTGCACCCAGTTTTC |
| CD24 | TTCTCCAAGCACCCAGCA | TGGAATAAATCTGCGTGGGTA |
| TGF-β | GCAGCACGTGGAGCTGTA | CAGCCGGTTGCTGAGGTA |
| β-actin | GAGCTGCGTGTGGCTCCC | CCAGAGGCGTACAGGGATAGCA |
